# Use of High-Dose Nebulized Colistimethate in Patients with Colistin-Only Susceptible *Acinetobacter baumannii* VAP: Clinical, Pharmacokinetic and Microbiome Features

**DOI:** 10.3390/antibiotics12010125

**Published:** 2023-01-09

**Authors:** Gennaro De Pascale, Gabriele Pintaudi, Lucia Lisi, Flavio De Maio, Salvatore Lucio Cutuli, Eloisa Sofia Tanzarella, Simone Carelli, Gianmarco Lombardi, Melania Cesarano, Veronica Gennenzi, Gabriella Maria Pia Ciotti, Domenico Luca Grieco, Brunella Posteraro, Maurizio Sanguinetti, Pierluigi Navarra, Massimo Antonelli

**Affiliations:** 1 Dipartimento di Scienze Dell’emergenza, Anestesiologiche e Della Rianimazione, Fondazione Policlinico Universitario A. Gemelli IRCCS, L.go A. Gemelli 8, 00168 Rome, Italy; 2 Dipartimento di Scienze Biotecnologiche di base Cliniche Intensivologiche e Perioperatorie, Universita’ Cattolica del Sacro Cuore, Rome, L.go F. Vito 1, 00168 Rome, Italy; 3 Section of Pharmcology, Department of Healthcare Surveillance and Bioethics, Catholic University Medical School, Fondazione Policlinico Universitario A. Gemelli IRCCS, L.go A. Gemelli 8, 00168 Rome, Italy; 4 Dipartimento di Scienze di Laboratorio e Infettivologiche, Fondazione Policlinico Universitario A. Gemelli IRCCS, L.go A. Gemelli 8, 00168 Rome, Italy; 5 Dipartimento di Scienze Mediche e Chirurgiche, Fondazione Policlinico Universitario A. Gemelli IRCCS, L.go A. Gemelli 8, 00168 Rome, Italy

**Keywords:** colistin, nebulization, ventilator-associated pneumonia, *Acinetobacter baumannii*

## Abstract

(1) Background: Colistin-only susceptible (COS) *Acinetobacter baumannii* (AB) ventilator-associated pneumonia (VAP) represents a clinical challenge in the Intensive Care Unit (ICU) due to the negligible lung diffusion of this molecule and the low-grade evidence on efficacy of its nebulization. (2) Methods: We conducted a prospective observational study on 134 ICU patients with COS-AB VAP to describe the ‘real life’ clinical use of high-dose (5 MIU q8) aerosolized colistin, using a vibrating mesh nebulizer. Lung pharmacokinetics and microbiome features were investigated. (3) Results: Patients were enrolled during the COVID-19 pandemic with the ICU presenting a SAPS II of 42 [32–57]. At VAP diagnosis, the median PaO_2_/FiO_2_ was 120 [100–164], 40.3% were in septic shock, and 24.6% had secondary bacteremia. The twenty-eight day mortality was 50.7% with 60.4% and 40.3% rates of clinical cure and microbiological eradication, respectively. We did not observe any drug-related adverse events. Epithelial lining fluid colistin concentrations were far above the CRAB minimal-inhibitory concentration and the duration of nebulized therapy was an independent predictor of microbiological eradication (12 [9.75–14] vs. 7 [4–13] days, OR (95% CI): 1.069 (1.003–1.138), *p* = 0.039). (4) Conclusions: High-dose and prolonged colistin nebulization, using a vibrating mesh, was a safe adjunctive therapeutic strategy for COS-AB VAP. Its right place and efficacy in this setting warrant investigation in interventional studies.

## 1. Introduction

Ventilator-associated pneumonia (VAP) caused by extensively drug-resistant (XDR) Gram-negative bacteria represent a clinical challenge for Intensive Care Unit (ICU) physicians, especially when old and potentially toxic drugs remain the only therapeutic armamentarium [1,2,3,4].

Despite the recent introduction of new antibiotics against difficult-to-treat bacteria, colistin is still used in the critically ill setting, especially as part of combination therapies [5]. This drug is a 50-years-old antibiotic; it is administered as intravenous colistimethate (CMS) and then undergoes extensive plasmatic hydrolysis. CMS is mainly metabolized by the renal route apart from the amount that is modified to colistin. This agent is cleared by extra-renal, yet unknown, mechanisms, but its plasmatic levels may increase in the presence of renal failure due to the reduced clearance and augmented conversion of CMS. Colistin use is limited by a low safety profile due to renal and neurotoxicity, especially when high dosages are required in order to obtain the pharmacokinetic/pharmacodynamic (PK/PD) targets: these concerns are particularly relevant when treating lung infections, as penetration of this molecule into the epithelial lining fluid (ELF) is minimal [6]. However, despite recent pharmacokinetic results suggesting its usefulness as adjunctive nebulization therapy for VAP treatment, current guidelines recommend against the use of inhaled antibiotics in the clinical practice, due to the paucity of available large-scale clinical data, the absence of well-defined indications in terms of dosages and nebulization practices, and the possible occurrence of pulmonary side-effects [7].

In light of that, the aim of this study is to describe the ‘real life’ clinical use of high-dose (5 MIU q8) nebulized CMS, administered by means of a vibrating mesh nebulizer, in a large cohort of hypoxemic critically ill patients with colistin-only susceptible (COS) *Acinetobacter baumannii* (AB) VAP. ELF pharmacokinetics and pulmonary microbiome features are also described in a subset of patients.

## 2. Methods

### 2.1. Study Setting and Design

This observational study prospectively included hospitalized patients across the two COVID-19 ICUs (75 beds) of the Fondazione Policlinico Universitario “A. Gemelli IRCCS” (Rome, Italy), between 1 March 2020 and 31 December 2021. Patients were eligible for inclusion if they were treated with high-dose nebulized colistin, for at least 48 h, due to a COS-AB VAP.

Patients received 5 MIU of CMS (Colimycine^®^, Sanofi, Paris, France) dissolved in 6 mL of saline solution by nebulization over 30 min using a vibrating-mesh nebulizer (Aeroneb Pro^®^, Aerogen, Galway, France) every 8 h. Solutions for nebulization were freshly prepared. During aerosol delivery, all patients were sedated and received assisted/controlled mechanical ventilation with a tidal volume of 6–8 mL/kg of predicted body weight and respiratory rates of 12–15 cycles/min. The humidifier was removed and the nebulizer was inserted near the Y-piece connector on the inspiratory arm

Electronic patient records and microbiology laboratory data were used to identify patients and to retrieve clinical data, microbiological results, and outcomes. In a subset of patients, pharmacokinetics of colistin in the ELF and lung microbiome analysis were also performed. The study was performed in accordance with the Declaration of Helsinki and was approved by the Ethics Committee of the Fondazione Policlinico “A. Gemelli IRCCS” (reference number ID3141). A written informed consent or proxy consent was waived, due to the observational nature of the study, according to committee recommendations. All data were anonymous and identified with an admission code number.

### 2.2. Definitions and Outcomes

VAP, septic shock, and acute kidney injury requiring continuous real replacement therapy were defined according to current recommendations. VAP was diagnosed in the presence of radiological and clinical signs consisting of a new and persistent infiltrate on the chest radiograph associated with two of the three following criteria: purulent tracheal aspirates, hyperthermia > 38 °C or hypothermia < 36 °C, and peripheral leukocytosis > 10,000/μL or < 1500/μL. A microbiological confirmation is required using tracheal aspirate ≥ 105 CFU/mL or broncho-alveolar lavage ≥ 10^4^ CFU/mL. VAP was defined as bacteremic when the microbiological diagnosis coincided with the same isolation in at least one blood culture in the absence of other specified sources of bacteremia [8]. Clinical cure was defined as the complete resolution of all signs and symptoms of the infection by the end of therapy, and an improvement or lack of progression of all abnormalities on chest radiographs was also required for VAP. Microbiological eradication was defined as the absence of the original pathogen from the culture of the specimens subsequently collected from the tracheobronchial tree [9]. Investigated outcomes were 28-day and 90-day mortality, clinical cure, microbiological eradication, post-VAP duration of hospital stay, ICU stay, and mechanical ventilation. Safety and adverse events (AE) were determined through the biochemical abnormalities documented in medical records according to the Department of Health and Human Services—Common Terminology Criteria for Adverse Events (DHHS-CTCAE version 3.0) classification. The severity of AE was graded from 1 to 5.

### 2.3. Pharmacokinetic Analysis

According to patients’ respiratory status, two micro-bronchoalveolar lavages (BALs) (40 mL of sterile 0.9% saline solution were blindly instilled through a telescopic catheter and immediately aspirated in a trap) were performed at steady state, before nebulization and 1 h after the end. ELF colistin concentration (COLELF) was calculated from BAL concentration (COLBAL) using urea as a dilution marker: COLELF = COLBAL X urea dilution index (plasma urea concentration/BAL urea concentration) [10].

Purification and separation of colistin and CMS were performed using and modifying the Gobin assay [11]. BAL samples were extracted by solid-phase extraction followed by evaporation to dryness and reconstitution in mobile phase. The chromatographic separation was carried out on an AQUITY UPLC C18 column. Polymyxin B was used as internal standard. The detection was performed on a triple quadrupole tandem mass spectrometer using multi-reaction monitoring via an electrospray ionization source with positive ionization mode.

### 2.4. Microbiological Analysis

AB isolates were identified by using matrix-assisted laser desorption ionization-time of flight mass spectrometry (MALDI-TOF MS). Minimum inhibitory concentrations (MICs) were determined by a Micronaut AST system-based BMD, VITEK 2 AST-N397 card, according to the manufacturer’s instructions. EUCAST (version 11.0, 2021) clinical breakpoints were used to interpret MICs.

The V3–V4 hypervariable regions of the 16S rRNA gene were amplified and sequenced using the Illumina MiSeq instrument as reported in De Pascale et al., 2021 [12]. After demultiplexing of the raw sequencing reads, FastQ sequences were analyzed according to the QIIME 2 (Quantitative Insights into Microbial Ecology 2) bioinformatics pipeline and analyzed by using RStudio and phyloseq package [13,14,15]. Sequencing reads have been submitted to the NCBI Sequence Read Archive (PRJNA693784).

### 2.5. Statistical Analysis

Clinical data analysis was performed using SPSS Statistical Software version 28.01.0 (IBM, Armonk, NY, USA), whereas data were graphed using GraphPad Prism version 6.0 (GraphPad Software, San Diego, CA, USA). Differences between groups for continuous data were assessed using either Student’s *t*-test (normally distributed) or the Mann–Whitney U-test (non-normally distributed), whereas those for categorical data were assessed using the chi-square test or Fisher’s exact test as appropriate. Odds ratios and 95% confidence intervals were calculated. Variables with a *p* value <0.1 in univariable analysis were included in multivariable analyses, which were conducted using stepwise logistic regression.

## 3. Results

### 3.1. Population Characteristics and Treatment

During the study period, 1386 patients were admitted to the ICU, and >60% received invasive mechanical ventilation (IMV). One hundred thirty-four developed COS-AB VAP and were treated with nebulized high-dose colistin for a median duration of 10 days [5,6,7,8,9,10,11,12,13]. Intravenous colistin was administered with a 9 MIU loading dose followed by 5.5 MIU q12; during CRRT, the dosage was increased to 6.75 MIU q12. Patients’ characteristics are shown in Table 1: the median Simplified Acute Physiology Score (SAPS II) was 42 [32–57], and most patients were affected by chronic cardiovascular diseases (67.9%), chronic obstructive pulmonary disease (17.2%), and diabetes (22.4%). VAP developed after a median of 9 [6–15] days of MV with a PaO_2_/FiO_2_ far from 150 mmHg and a high rate of septic shock (40.3%). One quarter of the patients had concomitant bacteremia and was treated with an intravenous combination of cefiderocol (2 g q8–6 h), tigecycline (100 mg of q12 after a 200 mg loading dose) or fosfomycin (8 g q8). All patients were treated with intravenous colistin for a median duration of 8 [3–11] days, according to clinical status.

### 3.2. Clinical Outcomes and Microbiological Findings

During 1376 days of high-dose colistin nebulization, we did not observe any drug-related adverse events, and not even aerosolization was interrupted due to bronchospasm, Y-piece obstruction, mucus plugs, or respiratory deterioration. In the absence of a control arm, we could only document observational, crude outcome measures results: 81 (60.4%) healed from VAP, and 54 (40.3%) underwent microbiological eradication, with a 28-day and 90-day mortality rate of 50.7% and 58.2%, respectively. The durations of the MV and ICU stay after VAP diagnosis were 12 [7.5–19] days and 15 [9–27.75] days, respectively.

Univariate analysis of microbiological eradication predictors were: younger age, lower SAPS II value, higher PaO_2_/FiO_2_, absence of septic shock, and longer durations of nebulized and intravenous colistin therapy. Multivariable logistic regression confirmed only SAPS II (OR (95%CI) 0.963 (0.940–0.986), *p* = 0.002), PaO_2_/FiO_2_ (OR (95%CI) 1.008 (1.001–1.015), *p* = 0.034), and duration of colistin nebulization (OR (95%CI) 1.069 (1.003–1.138), *p* = 0.039) as independent predictors of microbiological eradication (Table 2).

**Figure 1:**
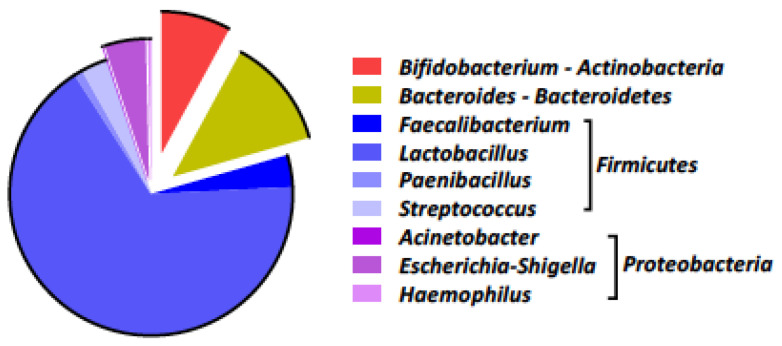
Lung microbiome composition of a representative patient with microbiological eradication.

Lung microbiome analysis of surveillance BAL, at the phylum and genus level, was performed in a patient with microbiological eradication after 12 days of nebulized therapy (Figure 1). It showed a clear predominance of *Firmicutes* (*Faecalibacterium*, *Lactobacillus*, *Paenibacillus,* and *Streptococcus*), compared with a marked reduction in *Proteobacteria*, especially *Acinetobacter*, *Bacteroidetes,* and *Actinobacteria*.

### 3.3. Pharmacokinetic Findings

**Figure 2:**
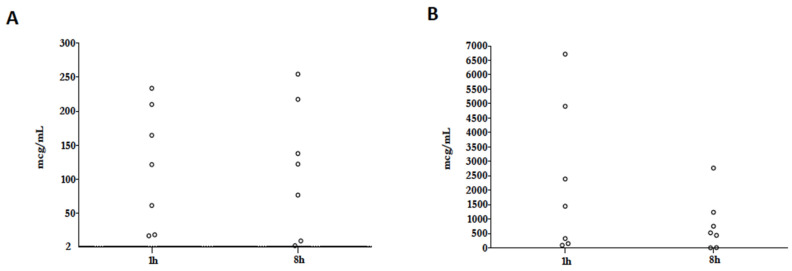
Colistin (**A**) and colistimethate (**B**) ELF concentrations. Data available from 7 patients’ ELF (epithelial lining fluid).

We investigated colistin and colistimethate (CMS) ELF concentration in seven patients after 72 h of nebulized therapy. One hour after nebulization, the median colistin and CMS concentrations were 121.7 [40.1–143.1] mcg/mL and 1445.3 [236.2–1918.2] mcg/mL, respectively. Twelve hours after nebulization, the median colistin and CMS concentrations were 122.6 [43.3–130] mcg/mL and 522.3 [222.3–636.5] mcg/mL, respectively. Colistimethate underwent extensive pulmonary hydrolysis to colistin, whose ELF concentration was far from the median MIC (1 mcg/mL) of isolated *Acinetobacter baumannii* (Figure 2A,B).

## 4. Discussion

In a cohort of 134 patients with COS-AB VAP, treated with inhaled colistin at a dose of 5 MIU q8, delivered through a vibrating mesh nebulizer, we obtained a clinical cure of 60.4%, without observing any pulmonary adverse events. Longer therapy duration was associated with higher rates of microbiological eradication, observing very high colistin ELF concentrations and deep changes in lung microbiome communities.

Current guidelines recommend avoiding nebulized antibiotics, including colistin, either as adjunctive or substitution therapy, for the treatment of lower-respiratory-tract infections, especially in patients with severe hypoxemia [7]. Such a position derives from a weak evidence level of their efficacy and the high potential for underestimated risks of side-effects. However, the availability of new, high-performing, nebulizers, along with the recent adoption of high-dose aerosolizing strategies, have raised scientific and clinical interest on the use of nebulized colistin in a specific sub-group of patients, such as COS-AB VAP [16,17,18,19].

There is pre-clinical evidence to support vibrating-mesh nebulizers, over jet and ultrasonic ones. Although slightly larger, vibrating-mesh aerosol particles remain below 5 microns, reaching the distal lung, with substantially reduced nebulization time and residual volume [20,21]. Alveolar deposition is further increased by the application of specific aerosolization strategies, including the nebulizer position 15 cm before the Y piece, the use of continuous aerosolization rather than breath-synchronous, the adoption of specific respiratory circuits to avoid sharp angles and turbulences, and controlled ventilatory modalities with constant inspiratory flow. On top of that, high-dose drug nebulization (i.e., 5 MIU of CMS q 8 h) allows very high tissue concentrations with a residual diffusion in the systemic compartment and kidney elimination [17]. All these concepts were first introduced by a seminal paper of the French Nebulized Antibiotics Study Group [22], where the use of 5 MIU q8 h, using a vibrating plate nebulizer, allowed a similar clinical cure rate in 44 patients with multidrug-resistant AB and *Pseudomonas aeruginosa*, compared with 122 controls treated with intravenous antibiotics. However, up to now, only small case series, mainly focusing on pharmacokinetics, confirmed the feasibility of the above therapeutic approach [23,24].

In our cohort of patients, we did not observe any side-effects related to CMS aerosolization. Similarly, Benitez-Cano et al., in 27 patients undergoing high-dose nebulization (9–15 MIU/day), did not report any episode of bronchospasm, although the majority of patients were already receiving bronchodilators [25], and a meta-analysis on 373 patients, using different CMS dosages and nebulizer types, showed a 33% reduction in the rate of renal failure, with 3% of neuromuscular toxicity and 2% of bronchospasm [26]. In addition, the observed safety profile may be further improved by correct CMS dilution, which requires a 6 mL volume of normal saline for the delivery of 5 MIU dosage in about 30 min [23].

From a pharmacokinetic standpoint, CMS nebulization may overcome its negligible ELF penetration after intravenous administration [6]. Although many factors may interfere with the reliability of the detected lung antibiotics concentration, including the diagnostic technique, the binding to inflammatory molecules, and the heterogeneity of lung aeration, it is widely accepted that CMS nebulization is the only strategy to obtain alveolar levels above MIC for mostly Gram-negative MDR pathogens [27]. When treating VAP due to COS bacteria, a correct drug dose and nebulizer strategy should be recommended: in 20 patients undergoing 1 MIU nebulization, through jet and ultrasonic machines, colistin ELF concentrations were above 10 mcg/mL only in 25% of samples collected at 1 h after the aerosol [28]. Conversely, following 24 h of dosing of 3 MII and 5 MIU nebulized CMS, using vibrating meshes, trough-predicted colistin levels were 120.4 mcg/mL and 200.7 mcg/mL, respectively, in ten patients with MDR VAP [24]. Similar to the above results, in our patients, we observed a marked and sustained hydrolysis of CMS to colistin, ensuring very high ELF concentrations in almost all samples at both 1 and 12 h after drug delivery.

Given the abovementioned heterogeneity in current published investigations, it is clear why we do not have robust evidence on the efficacy of nebulized CMS as a therapeutic strategy for difficult-to-treat VAP. Interestingly, in a recent observational study on 326 patients with COS Gram-negative VAP, the use of aerosolized CMS, although at a dosage of 2 MIU q 8 h through a jet nebulizer, was associated with a significantly lower DAY-14 clinical failure rate, without affecting mortality [29].

Further, we do not have data on the optimal duration of nebulized therapy, although it is reasonable that longer courses (8–14 days) may be required to definitely eradicate the infection, as recently shown in a well-conducted meta-analysis of 11 randomized trials and 1210 patients [19]. Thus, it is not surprising that in our cohort, the duration of inhaled CMS was an independent predictor of microbiological eradication, observing such high ELF colistin concentrations and deep lung microbiome architecture changes in some illustrative patients.

This paper has some limitations. First, its design is purely observational without a control arm, so we cannot draw any conclusion on the real clinical efficacy of inhaled CMS, in comparison with other combination strategies including new molecules. Second, we investigated the colistin pulmonary pharmacokinetic and lung microbiome in an exemplifying subset of patients. Finally, part of the patients also received combination intravenous treatment, whose contribution to the clinical observed results is difficult to ascertain.

However, this the largest study where the clinical use of high-dose inhaled CMS through a vibrating-mesh nebulizer has been investigated, along with the evaluation of ELF colistin pharmacokinetics and the lung microbiome in a selected subgroup of patients.

## 5. Conclusions

High-dose and prolonged colistin nebulization using a vibrating mesh was a safe adjunctive therapeutic strategy for COS-AB VAP. Its right place and efficacy in this setting warrant investigation in future interventional studies.

## Figures and Tables

**Table 1:** Baseline characteristics, VAP presenting features, and outcomes of 134 enrolled patients.

**Baseline Characteristics**	
Age	66 [58–73]
Gender (male)	103 (76.9)
SAPS II	42 [32–57]
Hypertension	91 (67.9)
IHD	22 (16.4)
CHD	5 (3.7)
COPD	23 (17.2)
Cerebral Vasculopathy	11 (8.2)
Diabetes	30 (22.4)
CKD	14 (10.4)
Immunosuppression	10 (7.5)
**VAP Presenting Features**	
Pre-VAP Hospital LOS (days)	16 [12.5–24]
pre-VAP ICU LOS (days)	13 [8–18]
pre-VAP MV (days)	9 [6–15]
PaO_2_/FiO_2_	120 [100–164]
Septic Shock	53 (40.3)
AKI requiring CRRT	18 (14.2)
BSI	33 (24.6)
MIC * mcg/mL	1 [0.5–1]
Aerosol Colistin days	10 [5–13]
Intravenous Colistin days	8 [3–11]
**Outcome measures**	
28-day mortality	68 (50.7)
90-day mortality	78 (58.2)
Clinical cure	81 (60.4)
Microbiological eradication	54 (40.3)
Post-VAP Hospital LOS (days)	21 [10–46.5]
Post-VAP ICU LOS (days)	15 [9–27.75]
Post-VAP MV (days)	12 [7.25–19]
Aerosol adverse events	0 (0)

Categorical variables are expressed in count and percentage; continuous variables are expressed in median and interquartile range. * MIC values available for 79 patients. Abbreviations: AKI, acute kidney injury; BSI, bloodstream infection; CHD, chronic heart disease; CKD, chronic kidney disease; COPD, chronic obstructive pulmonary disease; CRRT, continuous renal replacement therapy; FiO_2_, inspired O_2_ fraction; ICU, intensive care unit; IHD, ischemic heart disease; LOS, length of stay; MIC, minimum inhibitory concentration; MV, mechanical ventilation; PaO_2_, arterial O_2_ pressure; SAPS II, Simplified Acute Physiology Score; VAP, ventilator associated pneumonia.

**Table 2:** Univariate and multivariate analysis of factors associated with AB microbiological eradication.

Variables	No. % of Patients		Univariate Analysis		Adjusted Analysis	
	AB Eradication (*n* = 55)	AB Persistence (*n* = 79)	*p* Value	OR (95%CI)	*p* Value	OR (95%CI)
Demographics and comorbidities						
Age	61 [52.5–67.25]	69 [60–74.25]	0.002	0.949 (0.918–0.981)	0.127	0.972 (0.936–1.008)
Gender (male)	42 (76.36)	61(77.22)	0.752	0.864 (0.35–2.136)	-	-
SAPS II	37.5 [25.5–50.25]	45 [35–62]	<0.001	0.961 (0.939–0.984)	0.002	0.963 (0.940–0.986)
Hypertension	35 (63.6)	56 (70.88)	0.377	0.719 (0.345–1.496)	-	-
IHD	6 (10.91)	16 (20.25)	0.157	0.482 (0.176–1.324)	-	-
CHD	1 (1.81)	4 (5.06)	0.35	0.347 (0.038–3.194)	-	-
COPD	9 (16.36)	14 (17.72)	0.838	0.908 (0.363–2.276)	-	-
Cerebral Vasculopathy	4 (7.27)	7 (8.86)	0.742	0.807 (0.224–2.901)	-	-
Diabetes	12 (21.82)	18 (22.78)	0.895	0.946 (0.413–2.165)	-	-
CKD	8 (14.55)	6 (7.59)	0.203	2.071 (0.676–6.348)	-	-
Immunosuppression	3 (5.45)	7 (8.86)	0.465	0.593 (0.147–2.403)	-	-
VAP Presenting Features and Treatment						
PaO_2_/FiO_2_	131.5 [108.5–180]	114.5 [90–151]	0.006	1.009 (1.003–1.016)	0.034	1.008 (1.001–1.015)
Septic Shock	17 (30.91)	36 (45.57)	0.089	0.534 (0.259–1.101)	0.919	0.956 (0.401–2.277)
CRRT	6 (10.91)	12 (15.19)	0.466	0.677 (0.237–1.932)	-	-
Concomitant BSI	14 (25.45)	19 (24.05)	0.919	1.042 (0.469–2.315)	-	-
Nebulized Colistin days	12 [9.75–14]	7 [4–13]	0.012	1.075 (1.016–1.137)	0.039	1.069 (1.003–1.138)
Intravenous Colistin days	10 [6–12]	6 [2.75–10]	0.057	1.065 (0.998–1.137)	0.756	1.014 (0.928–1.109)

Categorical variables are expressed in count and percentage; continuous variables are expressed in median and interquartile range. Abbreviations: AB: *Acinetobacter baumannii*; VAP: Ventilator-Associated Pneumonia; SAPS II: Simplified Acute Physiology Score II; IHD: Ischemic Heart Disease; CHD: Chronic Heart Disease; COPD: Chronic Obstructive Pulmonary Disease; CKD: Chronic Kidney Disease. LOS: Length Of Stay; ICU: Intensive Care Unit; CRRT: Continuous Renal Replacement Therapy; BSI: Blood Stream Infection; IMV: Invasive Mechanical Ventilation.

## Data Availability

The data presented in this study are available on reasonable request from the corresponding author.
